# Thalamo-cortical mechanisms of the observed specific changes in frontal and occipital EEG rhythms during propofol-induce sedation

**DOI:** 10.1186/1471-2202-16-S1-P232

**Published:** 2015-12-18

**Authors:** Meysam Hashemi, Axel Hutt, Jamie Sleigh

**Affiliations:** 1 I NRIA CR Nancy - Grand Est, Villers-les-Nancy, France; 2 Department of Anaesthetics, Waikato Hospital, Hamilton, New Zealand

## 

**Figure 1:**
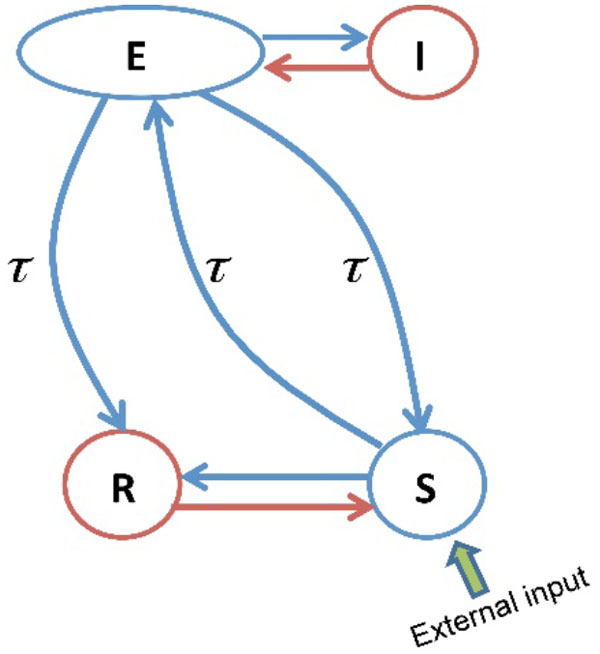
**Schematic of a thalamo-cortical module**. The blue arrows indicate excitatory connections and the red arrows represent inhibitory connections. The symbols E, I, S and R denote the cortical excitatory and inhibitory neurons, thalamo-cortical relay, and thalamic reticular neurons, respectively. In addition the connections between cortex and thalamus are associated with the same time delay τ.

Although general anesthesia is widely used in today's medical surgery, its precise underling mechanism is not yet clear. For clinically relevant concentration of propofol specific changes in electroencephalogram (EEG) rhythms can be observed experimentally. These characteristic changes comprised increased activity in the delta (0.5-4) Hz and alpha (8-13) Hz frequency bands over the frontal head region, but increased delta and decreased alpha activity over the occipital region [1]. The work model aims to understand the mechanisms underlying these specific changes in EEG power spectrum using a neuronal population model of a single thalamo-cortical module (Figure 1) based on a recently developed neural field model of anesthetic action [2,3]. The model reproduces well the certain changes observed experimentally in EEG rhythms over both frontal and occipital electrodes during propofol anesthesia sedation.

The power spectral analyses reveal that the alpha power originates from the cortico-thalamic relay interaction, which is associated with a constant time delay around the inverse of peak frequency in alpha band. It is shown that as the concentration of propofol increases, dependent on the potential values of the resting state of the system, it causes an increase or decrease in the gains function within the thalamo-cortical loop what then results in an increase or decrease in the spectral power in the alpha band over frontal and occipital regions, respectively. The model indicates the importance of multiple resting states in brain activity. Moreover our findings demonstrate that the emergence of delta power results from the increased GABAergic inhibition into the thalamo-cortical system.

Our results reveal that the specific observed changes in EEG rhythms can be reproduced with and without the propofol effect in cortical cells. This finding points out the importance of thalamus for neural effects under anesthesia sedation and simplifies the model under study. By reducing the dimensionality of the model we are able to obtain some inequality conditions for the stability of the system. In addition, the analytical tractability of the model allows us to obtain further insight into the mechanisms underlying the characteristic spectral features seen during anesthesia sedation.
